# A pyroptosis-related gene signature for prognosis prediction in hepatocellular carcinoma

**DOI:** 10.3389/fonc.2023.1085188

**Published:** 2023-03-27

**Authors:** Yongwei Chen, Yanyun Zhu, Yuanmei Dong, Huizi Li, Chumeng Gao, Guoqiang Zhu, Xiao Mi, Chengcheng Li, Yu Xu, Guoqiang Wang, Shangli Cai, Yusheng Han, Chunwei Xu, Wenxian Wang, Shizhong Yang, Wenbin Ji

**Affiliations:** ^1^ Faculty of Hepato-Pancreato-Biliary Surgery, Chinese PLA General Hospital, Beijing, China; ^2^ Senior Department of Oncology, The Fifth Medical Center of PLA General Hospital, Beijing, China; ^3^ Department of Nutrition, PLA Rocket Force Characteristic Medical Center, Beijing, China; ^4^ Jingnan Medical District, PLA General Hospital, Beijing, China; ^5^ Medical Department, Burning Rock Biotech, Guangzhou, Guangdong, China; ^6^ Institute of Basic Medicine and Cancer (IBMC), Chinese Academy of Sciences, Hangzhou, China; ^7^ Department of Clinical Trial, The Cancer Hospital of the University of Chinese Academy of Sciences (Zhejiang Cancer Hospital), Hangzhou, China; ^8^ Hepato-Pancreato-Biliary Center, Beijing Tsinghua Changgung Hospital, School of Clinical Medicine, Tsinghua University, Beijing, China

**Keywords:** pyroptosis, hepatocellular carcinoma, prognosis, antiangiogenic therapy, risk score

## Abstract

**Introduction:**

Hepatocellular carcinoma (HCC) is one of the most invasive cancers with a low 5-year survival rate. Pyroptosis, a specialized form of cell death, has shown its association with cancer progression. However, its role in the prognosis of HCC has not been fully understood.

**Methods:**

In our study, clinical information and mRNA expression for 1076 patients with HCC were obtained from the five public cohorts. Pyroptotic clusters were generated by unsupervised clustering based on 40 pyroptosis-related genes (PRGs) in the TCGA and ICGC cohort. A pyroptosis-related signature was constructed using least absolute shrinkage and selection operator (LASSO) regression according to differentially expressed genes (DEGs) of pyroptotic clusters. The signature was then tested in the validation cohorts (GES10142 and GSE14520) and subsequently validated in the CPTAC cohort (n=159) at both mRNA and protein levels. Response to sorafenib was explored in GSE109211.

**Results:**

Three clusters were identified based on the 40 PRGs in the TCGA cohort. A total of 24 genes were selected based on DEGs of the above three pyroptotic clusters to construct the pyroptotic risk score. Patients with the high-risk score showed shorter overall survival (OS) compared to those with the low-risk score in the training set (P<0.001; HR, 3.06; 95% CI, 2.22-4.24) and the test set (P=0.008; HR, 1.61; 95% CI, 1.13-2.28). The predictive ability of the risk score was further confirmed in the CPTAC cohort at both mRNAs (P<0.001; HR, 2.99; 95% CI, 1.67-5.36) and protein levels (P<0.001; HR, 2.97; 95% CI 1.66-5.31). The expression of the model genes was correlated with immune cell infiltration, angiogenesis-related genes, and sensitivity to antiangiogenic therapy (P<0.05).

**Discussion:**

In conclusion, we established a prognostic signature of 24 genes based on pyroptosis clusters for HCC patients, providing insight into the risk stratification of HCC.

## Introduction

Worldwide, primary liver cancer is the sixth most prevalent malignancy and the third leading cause of cancer death, with approximately 905,677 new cases and 830,180 deaths annually (1). Hepatocellular carcinoma (HCC) is the most common type of all primary liver cancer, accounting for 75%–85% of cases (2). At present, surgical resection is still the most effective treatment for HCC when diagnosed at an early stage, however, 70% of patients with HCC suffer recurrence or metastasis within 5 years after surgery (3). Despite significant advances in comprehensive HCC treatment, such as surgery, chemotherapy, radiotherapy, targeted therapy, and immunotherapy, the prognosis of patients with HCC remains poor, and the 5 years survival rate of HCC is only 5%-30% (4, 5). The poor prognosis of HCC may be due to its extreme heterogeneity and limited molecular treatment targets (6). There is an urgent need to develop novel prognostic signatures and therapeutic targets to predict survival and to outline individualized treatment plans for HCC patients.

Pyroptosis is a kind of programmed cell death (PCD) mediated by the Gasdermin family (GSDMs) including *GSDMA, GSDMB, GSDMC, GSDMD, GSDME*, and *DFNB59 (* 7). Upon cleavage by activated caspase-1/4/5/11 or granzyme proteases, the N-terminal of GSDMs oligomerizes in membrane form pores and results in cell membrane rupture (8). Pyroptosis was first discovered to play a crucial part in fighting against infection (9). Subsequently, emerging evidence revealed that inflammasome-mediated pyroptosis was linked to tumor development and immunity (10). Pyroptotic cells release a large number of immunogenic cellular contents including damage-associated molecular patterns (DAMPs) and inflammatory cytokines such as interleukin-1β (*IL-1β*) and *IL-18* and trigger inflammation, which may remodel the tumor immune microenvironment (TME) (11). Accumulated evidence indicated that pyroptosis may play a dual role in the pathogenesis of tumors and correlated with proliferation, migration, cell cycle, and drug resistance in multiple types of cancers (12). On one hand, the multiple inflammatory factors released during pyroptosis are closely related to tumorigenesis as well as chemotherapeutic resistance. On the other hand, as a type of cell death, pyroptosis can inhibit the occurrence and development of tumors and thereby serve as a potential target in tumor therapy (13). Increasing evidence has confirmed the important role of pyroptosis in multiple tumors including HCC (9). *GSDME* may function as a tumor suppressor gene in HCC as its expression is significantly lower in HCC cells and upregulating *GSDME* expression inhibited cell proliferation. In HCC, numerous studies have also shown the functions of pyroptosis and the prognostic value of pyroptosis-related genes (PRGs) in HCC progression (12–16). Hence, PRGs may become the potential biomarkers to predict the prognosis of HCC and provide guidance for treatment.

Considering that pyroptosis participates in the tumor pathogenesis and its role in the prognosis of HCC has not been fully understood, we performed a systemic analysis of PRGs in HCC and constructed a 24 PRG-related risk model to predict the prognosis of HCC based on differentially expressed genes (DEGs) of three pyroptotic clusters in the TCGA and ICGC training cohorts. The signature performed well in predicting HCC prognosis in the GEO validation datasets and the CPTAC cohort. And we also compared the molecular mechanisms in immunity and angiogenesis between the high- and low-risk groups. The predictive ability of the risk score was further confirmed at the protein level in CPTAC. These findings may provide novel insights into the prognosis and treatment of HCC.

## Patients and methods

### Data acquisition

Pyroptosis-related genes (PRGs) were obtained from msigdb (https://www.gsea-msigdb.org/) (**Supplementary Table 1**). The clinical and mRNA expression data in the training cohort are downloaded from The Cancer Genome Atlas (TCGA) and International Cancer Genome Consortium (ICGC) (https://xenabrowser.net/datapages/; https://dcc.icgc.org/), respectively. The validation cohorts including GSE10142 and GSE14520 datasets were downloaded from https://www.ncbi.nlm.nih.gov/geo/. Batch effects were removed with the R package (sva), and batch-removed results were shown in **Supplementary Figures 1A, B**. The CPTAC cohort was download from https://www.biosino.org/node/project/detail/OEP000321, and transcriptome and proteome were used in this study and the clinical information of patients is presented in **Supplementary Table 2**. The treatment cohort GSE109211 for sorafenib was also downloaded from the GEO database.

### Generation of PRGs-related risk model

Unsupervised clustering analysis based on 40 PRGs was done in the R package ConsensusClusterPlus (17). Differentially expressed genes (DEGs) analysis was identified with fold change>2 and p<0.05 using R package edgeR (18).

DEGs significantly associated with survival were analyzed using Cox regression of the R packages survminer and survival (19, 20). The R package glmnet was used to construct the risk model (21).

In the LASSO regression, a 5-cross-validation model with binomial deviance minimization criteria was implemented in the training set. The lambda with min was used for feature selection. Based on the selected gene markers, the predicted probability of each patient could be calculated using the following formula:


$$\text{Predicted probability =}\sum_{i}^{n}\left(Exp_{i}*Coef_{i}\right)$$

where n is the number of genes, Exp_i_ is the value of each gene, and Coef_i_ is the estimated regression coefficient of the selected genes.

The median value was used to divide patients into the high-risk and low-risk groups. The 1-, 2- and 3-year ROC curves and the ROC curves compared to predict the survival status with the R package survivalROC (22).

### Validation of the PRGs-related risk model

The prognostic performance of the PRG-related risk model was further validated in the external validation cohort (GSE10142 and GSE14520 datasets) and the CPTAC dataset. Patients are stratified into low- and high-risk groups using the median value as the cutoff. The independent prognostic value of the PRGs-related risk score was assessed using univariable and multivariable analysis, respectively. The clinical information of GSE10142 other than overall survival (OS) could not be downloaded from the public database, so the independence test of the risk score was not performed in GSE10142. The prediction efficiency of the nomogram was evaluated by calibration curves.

### Statistical analysis

Continuous variables were compared by Mann-Whitney *U* test or Kruskal-Wallis H-test and categorical variables were compared by Chi-Squared test or Fisher exact test. Principal Components Analysis (PCA) was performed by R package FactoMineR. Survival was estimated by Kaplan-Meier curves, with the *p* determined by a log-rank test. CIBERSORT (https://cibersort.stanford.edu/) was used to estimate the infiltration of immune cells based on mRNA data. KEGG (Kyoto Encyclopedia of Genes and Genomes) enrichment analysis of genes with DEGs and Gene Set Enrichment Analysis (GSEA) were performed using R package clusterProfiler. The non-supervised clustering of mRNA data was performed by the k-means method (R package, ConsensusClusterPlus). The area under curve (AUC) and 95% confidence interval (CI) were generated to evaluate the model performance. Immunohistochemical data of the proteins were downloaded from the Human Protein Atlas (HPA) database (https://www.proteinatlas.org/). Drug susceptibility prediction was performed by R package oncoPredict. The correlation between risk score and immune cells was evaluated using person correlation. P<0.05 was considered to be statistically significant and all p were two-sided. All the statistical tests were performed using R software, version 4.0.1 (R Foundation for Statistical Computing Vienna, Austria).

## Results

### The characteristics of pyroptosis-related genes

**Figure 1:**
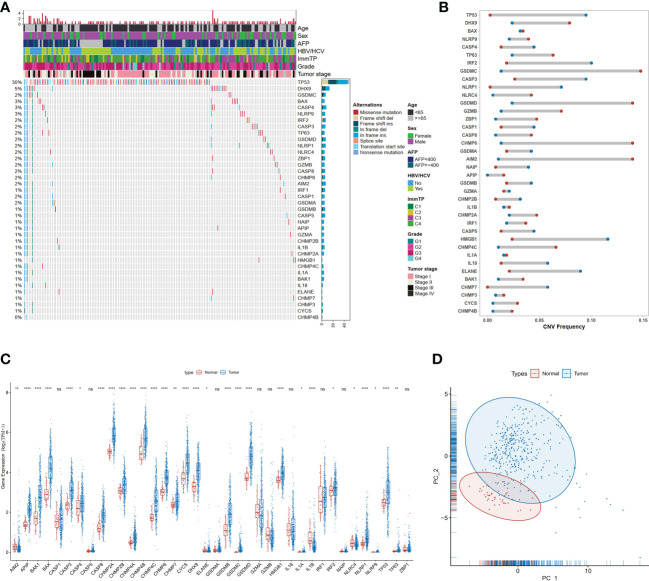
Genetic and transcriptional alterations of PRGs in HCC. **(A)** Mutation frequencies of 40 PRGs in the TCGA-LIHC dataset; **(B)** Frequencies of CNV gain (red dots), loss (blue dots), in HCC compared to normal tissue among PRGs; **(C)** Expression distributions of 40 PRGs between normal and HCC tissues, *P<0.05; **P<0.01; ***P<0.001; ****P<0.0001; "ns" represents "not significant"; **(D)** Principal component analysis (PCA) analysis of RRGs.

The 40 core genes that regulate or mediate pyroptosis signaling have been identified previously (23) and were available in the Molecular Signatures Database (MSigDB) (**Supplemental Table 1**). Gene mutations of pyroptosis-related genes (PRGs) occurred in the majority of hepatocellular carcinoma samples (54.4%) (**Figure 1A**), implying a potential role of PRGs in tumorigenesis and development of HCC. The TP53 gene was the most commonly mutated (30%), whereas mutations in other genes (*DHX9*, *GSDMC*, etc.) were less frequent. Frameshift deletions were the most common type of mutation, followed by frameshift insertions and missense mutations. Moreover, most PRGs had CNV gain or loss (**Figure 1B**). *GSDMC* was the most amplified gene with a frequency of 14.8% in HCC patients, in contrast, *HMGB1* had the highest CNV loss with a frequency of 11.6%. In addition, the mRNA expression of 77.5% (31/40) PRGs was significantly different between tumor and normal tissues (**Figure 1C**). Most of PRGs displayed higher expression levels in tumor samples compared with normal controls except for *AIM2*, *IL1B*, and *NLRC4*, of which the mRNA expression was decreased in tumor samples. Meanwhile, PCA based on the mRNA expression of PRGs could well differentiate HCC samples from normal samples (**Figure 1D**), suggesting the abnormal expression of PRGs in hepatocellular carcinoma.

### Identification of pyroptotic clusters

**Figure 2:**
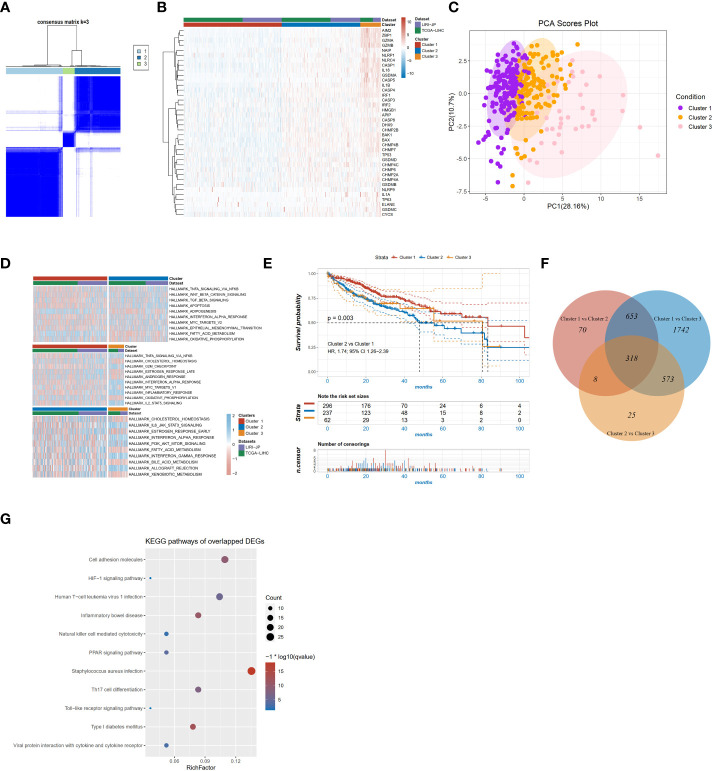
PRG clusters and biological characteristics of two distinct subtypes of samples divided by consistent clustering. **(A)** Consensus matrix heatmap defining three clusters (k=3) and their correlation area; **(B)** Heatmap of the mRNA expression of PRGs in the three clusters; **(C)** Principal components analysis of the three clusters; **(D)** GSVA of cancer hallmarks between three distinct clusters; **(E)** KM survival curve of the three clusters; **(F)** Venn diagram showing overlapping genes of the significant expressed genes between the three pyroptotic clusters; **(G)** KEGG enrichment analyses of DEGs among the three pyroptosis clusters.

To investigate the role of pyroptosis signaling in HCC, we further explored the role of PRGs in the prognosis of patients with HCC depending on different transcriptomic patterns. After eliminating the batch effect, two datasets derived from TCGA and ICGC were grouped as the training cohort. The patient characteristics were depicted in **Supplementary Table 2**. Unsupervised hierarchical clustering of transcriptome profiles of 40 PRGs identified three clusters (**Figure 2A**) in the training cohort. As delineated in the heatmap (**Figure 2B**), ranging from the cluster 1 to 3, the mRNA expression of PRGs was gradually increased. The principal components analysis also depicted the difference between the three clusters (**Figure 2C**). Gene Set Enrichment Analysis (GSEA) analysis on the signatures of cancer hallmarks confirmed that adipogenesis and oxidative phosphorylation were enriched in the cluster 1 but TNF-α signaling *via* NF-KB and WNT beta-catenin signaling were enriched in the cluster 2; conversely, the cluster 3 was characterized by genes in interferon-alpha response signaling (**Figure 2D**). Survival analysis found significant survival differences among the three clusters (log-rank *p* for trend = 0.004 **Figure 2E**). The cluster 2 exhibited shorter OS compared to the cluster 1 (p=0.003; HR, 1.74; 95% CI 1.26-2.39, **Figure 2E**). A two-by-two comparison of differentially expressed gene (DEG) analysis was performed and a total of 318 overlapped genes were found among the three pairs (**Figure 2F**). KEGG functional enrichment analysis was performed on the 318 overlapped DEGs, and significant pathways were partially presented in **Figure 2G**, such as the HIF-1 signaling pathway, natural killer cell-mediated cytotoxicity, and PPAR signaling pathway. Altogether, these results suggested that PRGs might play an important role in the prognosis of HCC.

### Construction and verification of the prognostic signature

**Figure 3:**
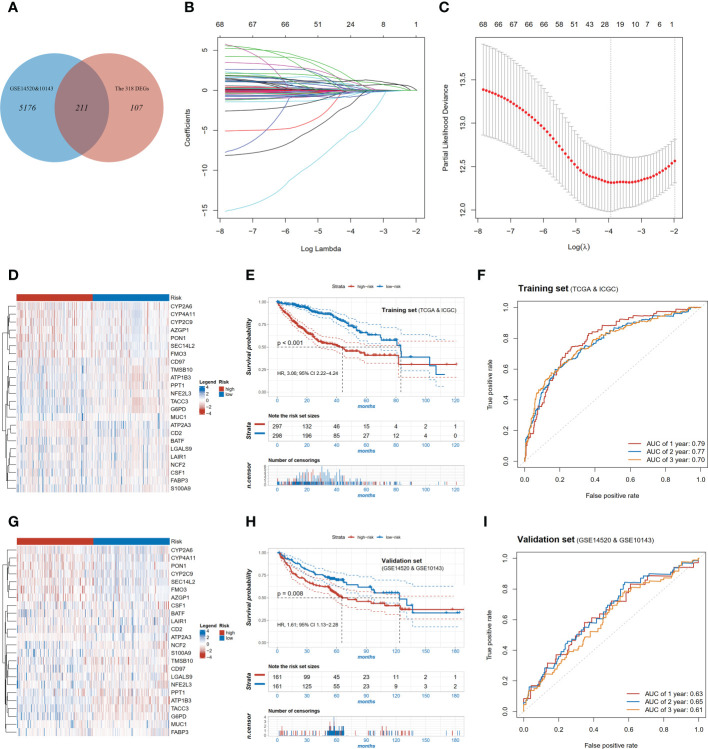
Construction and validation of the pyroptosis-related signature. **(A)** Venn diagram showing overlapping genes of DEGs and the genes detectable in GEO; **(B)** LASSO coefficient profiles of the 68 OS-associated genes; **(C)** Log Lambda value for LASSO regression; **(D)** Expression distributions of risk model genes between high and low risk patients in training set; **(E)** Kaplan-Meier survival curves of high and low risk patients in training set; **(F)** ROC curve of the risk model in training set; **(G)** Expression distributions of risk model genes between high and low risk patients in validation set; **(H)** Kaplan-Meier survival curves of high and low risk patients in validation set; **(I)** ROC curve of the risk model in validation set.

To specifically identify the prognosis-related core genes, a prognostic risk model for HCC was constructed based on DEGs using the LASSO regression method. We first selected 211 candidate genes which both existed in the training (TCGA and ICGC) and the validation cohorts (GSE14520 and GSE10143, **Figure 3A**). Among these genes, totally 68 genes were significant associated with OS by Cox proportional regression, and then a 24-gene risk model was constructed by LASSO regression (**Figures 3B, C**). Risk score = CYP2A6*(0.054) + PPT1*(0.4319) + G6PD*(0.6332) + CD97*(1.3631) + LGALS9*(-0.5509) + S100A9*(0.1446) + SEC14L2*(-0.2186) + NFE2L3*(0.0596) + FABP3*(-0.1861) + CD2*(-0.7902) + BATF*(0.7061) + PON1*(-0.336) + FMO3*(-0.3352) + CSF1*(0.2407) + CYP4A11*(0.1653) + ATP2A3*(-5.13) + MUC1*(-0.0063) + LAIR1*(-1.5441) + ATP1B3*(1.2079) + AZGP1*(0.0064) + NCF2*(1.0057) + CYP2C9*(-0.1876) + TMSB10*(-0.0666) + TACC3*(1.2139). Patients were divided into the high- and low-risk groups according to the median value of risk score. The transcriptomic profile of the selected 24 genes in the training set is illustrated in **Figure 3D**. Patients in the high-risk group had shorter OS compared with those in the low-risk group (HR=3.06, 95% CI 2.22-4.24, p<0.001, **Figure 3E**), and AUC value of 2-year survival was 0.77 in the training cohort (**Figure 3F**).

We further tested whether the risk score was an independent prognostic factor of HCC. In the univariable analysis, besides risk score, several other variables such as TNM stage, HBV or HCV infection, and TMB were also associated with the OS with HRs (95% CI) of 2.57 (1.90-3.47), 2.49 (1.82-3.40), 1.82 (1.36-2.44) and 1.91 (1.39-2.62), respectively (**Supplementary Tables 3, 4**). In the multivariable analysis, the association between the risk score and OS remained significant (HR=2.82, 95% CI 1.89-4.20, p<0.001, **Supplementary Tables 3, 4**). These results indicated that the risk score was associated with a better prognosis independent of TNM stage, HBV or HCV infection, and TMB.

To validate the performance of the 24-gene risk model, public datasets in GEO (GSE14520 and GSE10143) were collected together as the validation cohort after removing batch effect. The patients were also stratified into the high-risk and low-risk groups using the median value in the validation set. The transcriptomic profile of the selected 24 genes in the validation set is illustrated in **Figure 3G**. Consistently, patients with the high-risk score had shorter OS (HR=1.61, 95% CI 1.13-2.28, p=0.008, **Figure 3H**) in the validation set, and the AUC value of 2-year survival was 0.65 (**Figure 3I**). In the multivariable analysis, the risk score was also associated with OS (HR=1.70, 95% CI 1.08-2.69, p<0.001, **Supplementary Table 5**), which indicated to be an independent factor of prognosis in HCC.

**Figure 4:**
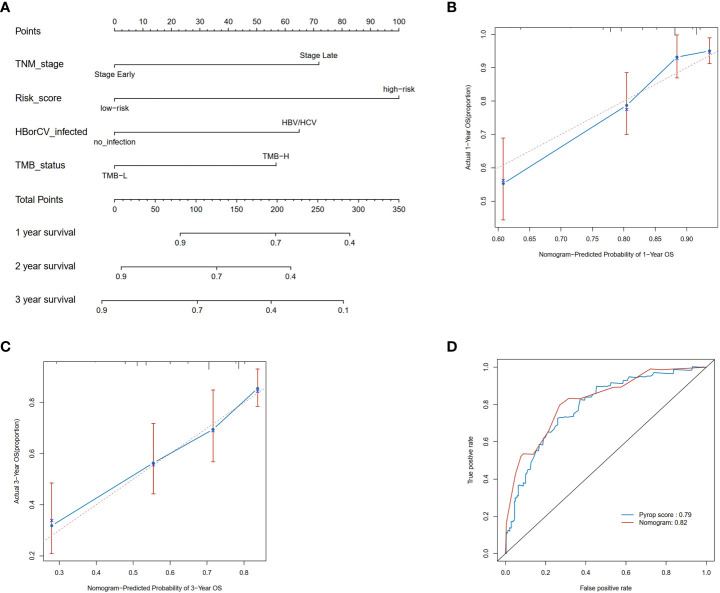
Construction and validation of a nomogram. **(A)** Nomogram for predicting the 1-, 2-, and 3-year OS of HCC patients in the TCGA cohort. **(B)** Calibration plots of the nomogram to predict OS at 1-year; **(C)** Calibration plots of the nomogram to predict OS at 3-year; **(D)** ROC curve of the nomogram and the risk model in the TCGA cohort at 1 year.

Next, a nomogram was constructed to predict the 1-, 2-, and 3-year OS rates in HCC patients integrating the risk score, tumor stage, HBV or HCV and TMB (**Figure 4A**). Calibration plots indicated that the nomogram had a good predictive power for 1-year and 3-year survival rates (**Figures 4B, C**). Compared to 1-year AUC value of 0.79 of pyroptosis risk score, the AUC value of the nomogram was up to 0.82 (**Figure 4D**). The nomogram further improved the performance and facilitated the clinical practice of the risk model.

### Validation of the prognostic signature in the CPTAC set at mRNA and protein levels

**Figure 5:**
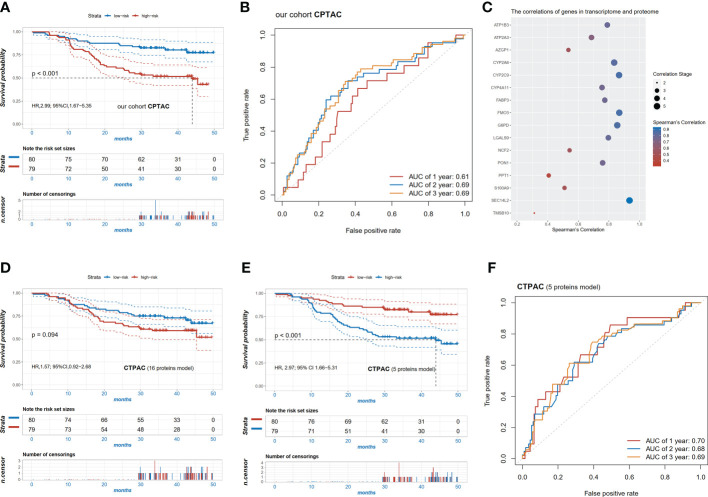
Validation of the pyroptosis-related signature in the cohort CPTAC. **(A)** Kaplan-Meier survival curves of high and low risk patients in the CPTAC cohort; **(B)** ROC curve in the CPTAC cohort; **(C)** the significant correlation of 16 signature genes (p<0.05) between transcriptome and proteome; **(D)** Kaplan-Meier survival curves of high and low risk patients based on the 16 protein levels in proteome; **(E)** Kaplan-Meier survival curves of high and low risk patients based on the 5 protein levels (r^2^>0.8) in the proteome; **(F)** ROC curve of the risk scores of the 5 proteins in the CPTAC cohort.

We further validated the prognostic value of the risk score in the CPTAC dataset at mRNA and protein levels, which consisted of the transcriptomic and proteomic data of 159 patients with HCC and corresponding clinical information. Consistent results were observed at the mRNA level that patients with the high-risk score had worse OS compared to those with the low-risk score (HR=2.99, 95% CI 1.67-5.35, p<0.001, **Figure 5A**) and AUC of 2-year survival was 0.69 (**Figure 5B**). Additionally, the robust performance was also observed in the multivariable analysis (HR=1.59, 95% CI 1.02-2.49, p=0.042, **Supplementary Table 6**).

To explore whether the risk models were also applicable at the protein level, the correlations between mRNA expression and protein expression of these candidate markers were examined. Finally, protein levels of 16 genes were significantly correlated with mRNA expression (p<0.05, **Figure 5C**). Using the same regression coefficients as the mRNA model, these proteins were also calculated for risk scores in the CPTAC cohort. Patients with the high-risk score tended to have a relatively shorter OS (HR=1.57, 95% CI 0.92-2.68, p=0.094) (**Figure 5D**). We further selected the top five most related proteins, characterized as Pearson correlation coefficient (R) above 0.8, including CYP2A6, CYP2C9, G6PD, FMO3, and SEC14L2. Unsurprisingly, the high-risk patients in the 5-proteins model also had worse OS compared with the low-risk group (HR=2.97, 95% CI 1.66-5.31, p<0.001, **Figure 5E**) and the model had a better predictive ability with a 2-year AUC of 0.68 (**Figure 5F**).

### The expression of the five proteins in HCC

**Figure 6:**
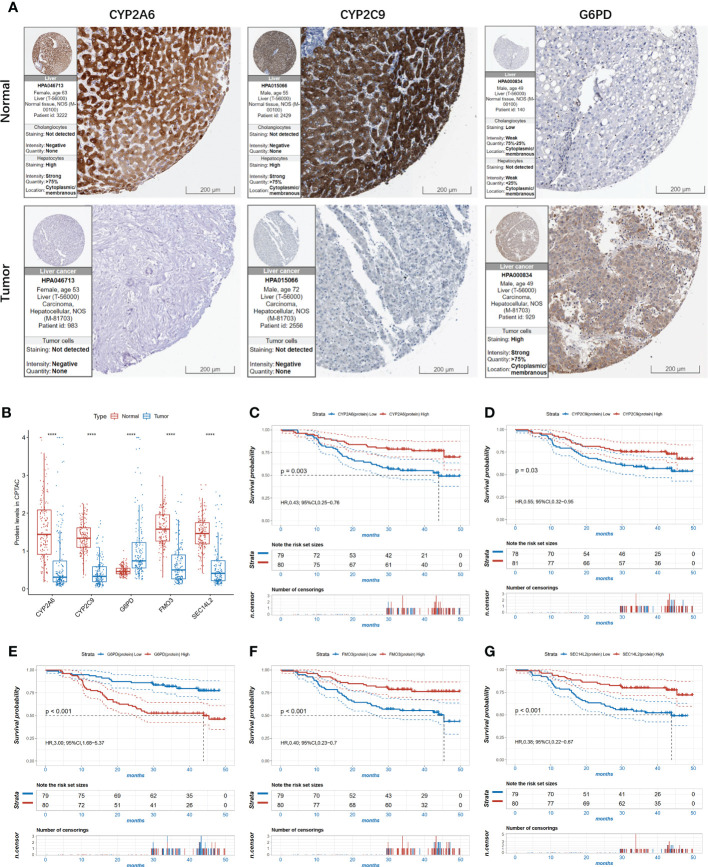
The expression level of the 5 proteins in HCC. **(A)** The immunohistochemistry (IHC) results from the Human Protein Atlas (HPA) was used to detect the protein level of three proteins in normal and tumor tissues; **(B)** The expression of the 5 proteins in proteomic level in normal and tumor tissues in the CPTAC cohort, ****P<0.0001.; **(C–G)** Kaplan-Meier survival curves of high and low expressed CYP2A6 **(C)**; CYP2C9 **(D)**; G6PD **(E)**; FMO3 **(F)**; SEC14 **(G)** in the CPTAC cohort.

To further confirm the importance of CYP2A6, CYP2C9, G6PD, FMO3, and SEC14L2, the Human Protein Atlas (HPA) database was used to compare their protein expression in normal and HCC tissues. As demonstrated in **Figure 6A**, the expression of CYP2A6 and CYP2C9 were relatively lower in tumor tissues, while the expression of G6PD in HCC tumors was higher in tumor tissues than in normal tissues. In the proteomic data of the CPTAC cohort, G6PD was also significantly highly expressed in HCC tumor tissues compared to normal tissues, while the remaining four proteins were significantly less expressed, consistent with the results of immunohistochemistry (**Figure 6B**). KM survival curves showed that HCC patients with higher CYP2A6, CYP2C9, FMO3, and SEC14L2 protein expressions had longer OS and those of higher G6PD had shorter OS in the CPTAC cohort (**Figures 6C–G**).

### Comparison of immunity and angiogenesis between the high and low-risk groups

**Figure 7:**
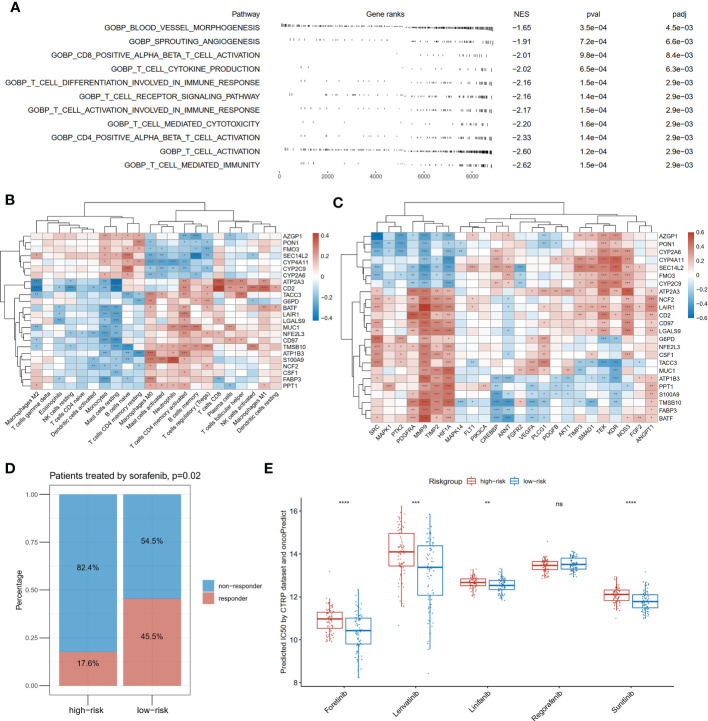
Differences in immunity and angiogenesis between high and low risk groups. **(A)** Two angiogenesis-related pathways and nine immune-related pathways were significantly enriched in the low-risk group by GSEA. NES means normalized enrichment score; **(B)** The heatmap of the correlation between the expression of the twenty-four genes constituting the risk model and the degree of immune infiltration; **(C)** The heatmap of the correlation between the expression of the twenty-four genes constituting the risk model and angiogenesis-related genes; **(D)** Comparison of response rates to sorafenib in high- and low-risk groups in GSE109211; **(E)** Drug sensitivity prediction of anti-angiogenic drugs except for sorafenib by oncoPredict based on the CTRP (Cancer Therapeutics Response Portal) database, *P<0.05; **P<0.01; ***P<0.001; ****P<0.0001.

To investigate the differences in physiological function between the high- and low-risk groups of patients, we compared the molecular mechanisms in immunity and angiogenesis. As shown in **Figure 7A**, blood vessel morphogenesis, sprouting angiogenesis and nine immunity-related pathways were significantly enriched in the patients with the low-risk score *via* GSEA. We next investigated the correlation between the expression of the 24 genes in the risk model and immune cell infiltration abundances and angiogenesis-related genes. The infiltration abundance of CD8-positive T cells was found to be significantly positively correlated with the expression of *ATP2A3*, *CD2*, *LGALS9*, and *NFE2L3* and negatively correlated with the expression of *FABP3* (p<0.05; **Figure 7B**). Activated natural killing cell infiltration was also correlated significantly positively with *CD2* and *TMSB10* expression and negatively with *SEC14L2* and *G6PD* (p<0.05; **Figure 7B**). Twenty-four angiogenesis-related genes were also applied in correlation analysis with genes in the risk model (**Figure 7C**). In terms of gene expression, *HIF1A* and *MMP9* were significantly correlated with all genes in the risk model (p<0.05; **Figure 7C**). In terms of *VEGFA*, one of the core targets of anti-angiogenic drugs was also found to be significantly positively correlated with *ATP2A3*, *G6PD*, *TACC3*, and *MUC1* and negatively correlated with *PPT1*, *S100A9*, *TMSB10*, *FABP3*, and *BATF* (p<0.05; **Figure 7C**).

At last, we applied the risk model to the GSE109211 cohort containing 67 cases with advanced HCC treated with sorafenib. In the low-risk group, the proportion who responded to sorafenib was 45.5%, higher than that (17.6%) in the high-risk group (p=0.02, **Figure 7D**). To compensate for the absence of other anti-angiogenic drugs in the treatment cohort, drug sensitivities were predicted by computation algorithm. The predicted IC_50_ of forentinib, Lenvatinib, linifanib, and sunitinib were all significantly lower in the low-risk group (**Figure 7E**).

## Discussion

HCC is the most common type of all primary liver cancer with a poor prognosis. Gene risk models based on specific cell activities, such as pyroptosis and immunization activities, have shown advantages in predicting the prognosis of HCC (12, 14–16, 24). A robust prediction model may help improve the outcomes of HCC patients. In our study, we first comprehensively analyzed the 40 PRGs in HCC and three different pyroptotic clusters were identified in the training set. Then the 318 DEGs of different pyroptotic clusters were used to establish a pyroptosis-related gene risk model to predict the prognosis of HCC in the training set. The 24-gene risk model was further tested in the validation set and a similar tendency was also seen in protein levels in the CPTAC cohort. Moreover, the low-risk group was associated with angiogenesis signaling and more sensitivity to anti-angiogenic drugs. In the study, we have included all the available public datasets with both mRNA expression and survival data, consisting of 1,076 patients from 5 cohorts. To our knowledge, this is the largest cohorts used for establishing a pyroptosis-related gene signature for prognosis prediction in HCC. To be noted, we also included the CPTAC-Liver dataset with both mRNA and protein data to validate our findings in both mRNA and protein levels.

Pyroptosis is a novel form of PCD, characterized by the formation of bubble-like morphology and releasing a great deal of inflammatory factors. Increasing evidence has implicated pyroptosis in the occurrence and development of tumors including HCC (13). The expression of *GSDME* in HCC cells was significantly lower than that in normal cells and upregulating *DFNA5/GSDME* expression inhibited cell proliferation, indicating that *GSDME* may be an anti-oncogene (13, 25). Besides, the expression of caspase-1, *IL-1β*, and *IL-18* was significantly lower in HCC tissues than those in adjacent normal tissues (26, 27). Among the pyroptosis-related inflammasomes, the *NLRP3* inflammasome has been studied in depth, and its influence in HCC pathogenesis has been extensively documented during the past several years. It is well established that the *NLRP3* inflammasome is released by pyroptotic hepatocytes and incorporated by adjoining cells, promoting inflammation and extracellular matrix deposition (28). The *NLRP3* inflammasome was reported to be downregulated in HCC tissue compared with normal liver and negatively correlated with pathological grades and clinical stage (29). Ding Y. et al. recently demonstrated that *NLRP3* expression was associated with the degree of B cell, CD4^+^ T cell, CD8^+^ T cell, neutrophils, and dendritic cell invasion (30). Besides, another study elaborated the molecular mechanism by which the *NLRP3* inflammasome initiated cancer cell death in HCC. 17β-estradiol (E2)-induced activation of the *NLRP3* inflammasome serves as a suppressor in HCC progression, as it triggers caspase 1-dependent pyroptotic cell death and inhibits protective autophagy *via* the E2/ERβ/AMPK/mTOR pathway (31). The block of *AIM2*—another inflammasome—was reported to induce mTOR-S6K1 pathway activation and thus promoted HCC progression (32). Another study revealed that *IRF1* increased CD8^+^ T cells, NK and NKT cells migration, and activated IFN-γ secretion in NK and NKT cells to induce tumor apoptosis through the CXCL10/CXCR3 paracrine axis in HCC (33). In addition, it has been reported that some antitumor drugs or molecules can trigger pyroptosis in HCC (34). Here in this research, we explored the genetic variations and expression of PRGs based on the TCGA cohort and global alterations in PRGs at the transcriptional and genetic levels in HCC. The majority of PRGs had CNV gain or loss, and most of them were upregulated in HCC patients at the transcriptional level. Moreover, PCA analysis based on PRGs successfully differentiates HCC samples from normal samples. These results indicated that PRGs may be involved in the prognostic prediction in HCC.

Three pyroptotic clusters were defined according to the expression of PRGs from 595 cases of HCC in the training cohort. The survival differs among different clusters, which indicates that PRGs might serve as a predictor for evaluating the clinical outcome of HCC. Then 318 DEGs among the three clusters were identified. After univariable Cox regression and LASSO regression analysis, a 24-gene prognostic model was constructed from DEGs. Patients with high expressions of *LGALS9*, *SEC14L2*, *FABP3*, *CD2*, *PON1*, *FMO3*, *ATP2A3*, *MUC1*, *LAIR1*, *CYP2C9*, and *MSB10* had a longer OS, while patients with high expression of *CYP2A6*, *PPT1*, *G6PD*, *CD97*, *S100A9*, *NFE2L3*, *BATF*, *CSF1*, *CYP4A11*, *ATP1B3*, *AZGP1*, *NCF2*, and *TACC3* had a poorer prognosis. In this signature, *FABP3*, *PON1*, *LAIR-1*, *CYP2A6*, *CYP2C9*, *BATF*, and *AZGP1*, et al. have been reported to be associated with survival in HCC (35–39). Among the above genes, FABP3 (fatty acid-binding protein) has been reported to be related involved in tumorigenesis and infiltrating immune cells, which can be a prognostic biomarker in non-small cell lung cancer, breast cancer, and esophageal cancer (40). Paraoxonase 1 (*PON1*), a calcium-dependent hydrolase protein synthesized mainly in the liver by hepatocytes, is a serum biomarker for the diagnosis of microvascular invasion (35). Low expression of PON1 was associated with poor survival in HCC patients (36). Leukocyte-associated immunoglobulin-like receptor-1 (*LAIR-1*) is an immune inhibitory receptor, high levels of *LAIR-1* expression are associated with poor cancer differentiation and overexpression of *LAIR-1* was significantly associated with worse overall survival in HCC (39). Decreased expression of *BATF2* and *AZGP1* was reported to be associated with a poor prognosis in HCC (37, 38). The risk model demonstrates high predictive accuracy and sensitivity, and its prognostic value was confirmed in the validation group. We then established a quantitative nomogram by integrating the risk score, tumor stage, HBV or HCV and TMB, which further improved the performance and facilitated the clinical use of the risk model. Altogether, our study further confirmed the prognostic value of these genes in HCC patients.

The predictive ability of the pyroptosis-related gene signature was further validated in the CPTAC cohort at both mRNA and protein levels. Interestingly, the risk score consisting of 5 proteins whose correlation with mRNA was above 0.8 also had a good predictive ability with a 2-year AUC of 0.68. The expression of the 5 proteins was associated with survival in HCC patients respectively. HCC patients with higher CYP2A6, CYP2C9, FMO3, and SEC14L2 protein expressions had longer OS and those of higher G6PD had shorter OS in the CPTAC cohort. To further confirm the importance of CYP2A6, CYP2C9, and G6PD in HCC, we used the HPA database to evaluate their protein expression levels in normal and HCC tissues. The expression of CYP2A6 and CYP2C9 was relatively lower in tumors tissues, but the G6PD expression level was higher in HCC tissues, which was consistent with the proteomic level in CPTAC. Cytochrome P450s (CYPs), a large group of enzymes that play crucial roles in the metabolism of endogenous and exogenous molecules, have been suggested that the expression of CYPs is very important for the management of cancer since these functionally associated enzymes might be involved in the development of HCC (41). *CYP2A6* is a CYP450 gene and has been reported its downregulation in tumor tissues is linked to worse overall survival and recurrence-free survival from hepatocellular carcinoma (41). *CYP2C9* was also reported downregulated in HCC tissue in part due to the de-differentiation of cancer cells and favorable factors in prognosis signature in HCC (42, 43). A previous study showed that *G6PD* (glucose-6-phosphate dehydrogenase) was highly expressed in HCC and was associated with poor prognosis (44). These results indicated that CYP2A6 and CYP2C9 might be protective factors, while G6PD might be risk factors in HCC, which improved the performance and facilitated the use of the risk model.

Moreover, GSEA found that blood vessel morphogenesis, sprouting angiogenesis, and immunity-related pathways were significantly enriched in patients with the low-risk score. The expression of the model genes was correlated with immune cell infiltration and the mRNA expression of angiogenesis-related genes. HCC is characterized by its aggressiveness and angiogenic capability; thus, the angiogenic factor *VEGF* is considered to be a target for HCC therapy (45). The multi‐kinase inhibitor sorafenib has been the global standard of care for advanced HCC for a decade with its anti‐angiogenic and anti-proliferative effects (46). Meanwhile, patients in the low-risk group showed a better response to sorafenib in the GSE109211 cohort. Interestingly, sorafenib has been reported to induce pyroptosis in macrophages and unleash the NK cell response in HCC (47). In addition, forentinib, Lenvatinib, linifanib, and sunitinib were predicted to be more sensitive in patients with the low-risk scoresrisk score, suggesting that PRGs are potentially associated with response to antiangiogenic therapy.

However, this work had several limitations. First, this is a retrospective study, in which the sample and transcriptomic data were derived from different platforms, and it may induce potential bias. However, the limitation of the retrospective setting can be greatly minimized by the large sample size (the five cohorts involving 1,076 patients). Moreover, the batch effect has been minimized by bioinformatics. Second, prospective studies and wet experiments underlying these pyroptosis-related genes and HCC prognosis are needed to confirm the reliability of the signature. Third, we used the HPA database (immunohistochemistry) to evaluate the expression of CYP2A6, CYP2C9, G6PD, FMO3, and SEC14L2 in normal and HCC tissues. However, future experiments are needed to further validate the findings in the HPA database. Forth, further experimental research including cell and molecular biology is needed to investigate the mechanisms.

In conclusion, we constructed and validated a 24 pyroptosis-related gene signature with robust utility for prognostication derived from the TCGA, ICGC, and GEO databases through enrichment, differential, and regression analyses. The signature was further validated at both the mRNA and protein levels in the CPTAC dataset and was connected with the response to antiangiogenic therapy. These observations provide a new idea for the molecular characterization of pyroptosis and the prognosis of HCC.

## Data availability statement

The datasets presented in this study can be found in online repositories. The names of the repository/repositories and accession number(s) can be found in the article/**Supplementary Material**.

## Author contributions

YC, YZ, SY, and WJ were the principal authors in conception and design of this study. YC, YZ, YD, HL, CG, GZ, and XM performed the data analysis and reviewed the literature. GW, SC, YH, SY, and WJ read and corrected the manuscript. All authors contributed to the article and approved the submitted version.

## References

[1] Sung H Ferlay J Siegel RL Laversanne M Soerjomataram I Jemal A. Global cancer statistics 2020: GLOBOCAN estimates of incidence and mortality worldwide for 36 cancers in 185 countries. CA: Cancer J Clin (2021) 71(3):209–49. doi: 10.3322/caac.21660 33538338

[2] Singal AG Lampertico P Nahon P. Epidemiology and surveillance for hepatocellular carcinoma: New trends. J hepatol (2020) 72(2):250–61. doi: 10.1016/j.jhep.2019.08.025 PMC6986771 31954490

[3] Ji F Fu S Guo Z Pang H Chen D Wang X. Prognostic significance of preoperative aspartate aminotransferase to neutrophil ratio index in patients with hepatocellular carcinoma after hepatic resection. Oncotarget (2016) 7(44):72276. doi: 10.18632/oncotarget.10848 27472390 PMC5342161

[4] Nault J-C Villanueva A. Intratumor molecular and phenotypic diversity in hepatocellular carcinoma. Clin Cancer Res (2015) 21(8):1786–8. doi: 10.1158/1078-0432.CCR-14-2602 25628398

[5] Allemani C Matsuda T Di Carlo V Harewood R Matz M Nikšić M. Global surveillance of trends in cancer survival 2000–14 (CONCORD-3): Analysis of individual records for 37 513 025 patients diagnosed with one of 18 cancers from 322 population-based registries in 71 countries. Lancet (2018) 391(10125):1023–75. doi: 10.1016/S0140-6736(17)33326-3 PMC5879496 29395269

[6] Liu LM Lin P Yang H Dang YW Chen G. Gene profiling of HepG2 cells following nitidine chloride treatment: An investigation with microarray and connectivity mapping. Oncol Rep (2019) 41(6):3244–56. doi: 10.3892/or.2019.7091 PMC6489000 30942464

[7] Liu X Xia S Zhang Z Wu H Lieberman J. Channelling inflammation: Gasdermins in physiology and disease. Nat Rev Drug Discovery (2021) 20(5):384–405. doi: 10.1038/s41573-021-00154-z 33692549 PMC7944254

[8] Lu X Guo T Zhang X. Pyroptosis in cancer: Friend or foe? Cancers (2021) 13(14):3620. doi: 10.3390/cancers13143620 34298833 PMC8304688

[9] Yu P Zhang X Liu N Tang L Peng C Chen X. Pyroptosis: Mechanisms and diseases. Signal transduction targeted Ther (2021) 6(1):1–21. doi: 10.1038/s41392-021-00507-5 PMC8005494 33776057

[10] Hou J Hsu J-M Hung M-C. Molecular mechanisms and functions of pyroptosis in inflammation and antitumor immunity. Mol Cell (2021) 81(22):4579–90. doi: 10.1016/j.molcel.2021.09.003 PMC8604761 34562371

[11] Frank D Vince JE. Pyroptosis versus necroptosis: Similarities, differences, and crosstalk. Cell Death Differentiation (2019) 26(1):99–114. doi: 10.1038/s41418-018-0212-6 30341423 PMC6294779

[12] Liu S Shao R Bu X Xu Y Ming S. Identification of the pyroptosis-related gene signature for overall survival prediction in patients with hepatocellular carcinoma. Front Cell Dev Biol (2021) 9:742994. doi: 10.3389/fcell.2021.742994 34820372 PMC8606528

[13] Xia X Wang X Cheng Z Qin W Lei L Jiang J. The role of pyroptosis in cancer: Pro-cancer or pro-”host”? Cell Death Dis (2019) 10(9):1–13. doi: 10.1038/s41419-019-1883-8 PMC6733901 31501419

[14] Liang N Li B Jia Z Wang C Wu P Zheng T. Ultrasensitive detection of circulating tumour DNA *via* deep methylation sequencing aided by machine learning. Nat BioMed Eng (2021) 5(6):586–99. doi: 10.1038/s41551-021-00746-5 34131323

[15] Zheng S Xie X Guo X Wu Y Chen G Chen X. Identification of a pyroptosis-related gene signature for predicting overall survival and response to immunotherapy in hepatocellular carcinoma. Front Genet (2021) 12:789296–. doi: 10.3389/fgene.2021.789296 PMC8678488 34925465

[16] Wang J Wang Y Steffani M Stöß C Ankerst D Friess H. Novel risk classification based on pyroptosis-related genes defines immune microenvironment and pharmaceutical landscape for hepatocellular carcinoma. Cancers (2022) 14(2):447. doi: 10.3390/cancers14020447 35053610 PMC8773536

[17] Wilkerson MD Hayes DN. ConsensusClusterPlus: A class discovery tool with confidence assessments and item tracking. Bioinformatics (2010) 26(12):1572–3. doi: 10.1093/bioinformatics/btq170 PMC2881355 20427518

[18] Robinson MD McCarthy DJ Smyth GK. edgeR: A bioconductor package for differential expression analysis of digital gene expression data. Bioinformatics (2010) 26(1):139–40. doi: 10.1093/bioinformatics/btp616 PMC2796818 19910308

[19] Therneau TM Grambsch PM. Modeling survival data: Extending the cox model. New York: Springer. (2000).

[20] Kassambara A Kosinski M Biecek P. Survminer: Drawing survival curves using 'ggplot2'. R package version 0.4.9. (2021). Available at: https://CRAN.R-project.org/package=survminer.

[21] Friedman J Hastie T Tibshirani R. Regularization paths for generalized linear models *via* coordinate descent. J Stat Software. (2010) 33(1):1–22. Available at: https://www.jstatsoft.org/v33/i01/. PMC2929880 20808728

[22] Patrick J. Heagerty and packaging by Paramita Saha-Chaudhuri. SurvivalROC: Time-dependent ROC curve estimation from censored survival data. R package version 1.0.3. (2013). Available at: https://CRAN.R-project.org/package=survivalROC.

[23] Liberzon A Birger C Thorvaldsdóttir H Ghandi M Mesirov JP Tamayo P. The molecular signatures database hallmark gene set collection. Cell systems (2015) 1(6):417–25. doi: 10.1016/j.cels.2015.12.004 PMC4707969 26771021

[24] Zheng Q Yang Q Zhou J Gu X Zhou H Dong X. Immune signature-based hepatocellular carcinoma subtypes may provide novel insights into therapy and prognosis predictions. Cancer Cell Int (2021) 21(1):1–14. doi: 10.1186/s12935-021-02033-4 34193146 PMC8243542

[25] Wang C-J Tang L Shen D-W Wang C Yuan Q-Y Gao W. The expression and regulation of DFNA5 in human hepatocellular carcinoma DFNA5 in hepatocellular carcinoma. Mol Biol Rep (2013) 40(12):6525–31. doi: 10.1007/s11033-013-2581-8 24154762

[26] Chen Y Qi H Wu F. Euxanthone exhibits anti-proliferative and anti-invasive activities in hepatocellular carcinoma by inducing pyroptosis: Preliminary results. Eur Rev Med Pharmacol Sci (2018) 22(23):8186–96. doi: 10.26355/eurrev_201812_16511 30556857

[27] Chu Q Jiang Y Zhang W Xu C Du W Tuguzbaeva G. Pyroptosis is involved in the pathogenesis of human hepatocellular carcinoma. Oncotarget (2016) 7(51):84658. doi: 10.18632/oncotarget.12384 27705930 PMC5356689

[28] Gaul S Leszczynska A Alegre F Kaufmann B Johnson CD Adams LA. Hepatocyte pyroptosis and release of inflammasome particles induce stellate cell activation and liver fibrosis. J Hepatol (2021) 74(1):156–67. doi: 10.1016/j.jhep.2020.07.041 PMC7749849 32763266

[29] Wei Q Mu K Li T Zhang Y Yang Z Jia X. Deregulation of the NLRP3 inflammasome in hepatic parenchymal cells during liver cancer progression. Lab Invest (2014) 94(1):52–62. doi: 10.1038/labinvest.2013.126 24166187

[30] Ding Y Yan Y Dong Y Xu J Su W Shi W. NLRP3 promotes immune escape by regulating immune checkpoints: A pan-cancer analysis. Int Immunopharmacol (2022) 104:108512. doi: 10.1016/j.intimp.2021.108512 35026655

[31] Wei Q Zhu R Zhu J Zhao R Li M. E2-induced activation of the NLRP3 inflammasome triggers pyroptosis and inhibits autophagy in HCC cells. Oncol Res (2019) 27(7):827–34. doi: 10.3727/096504018X15462920753012 PMC7848400 30940293

[32] Ma X Guo P Qiu Y Mu K Zhu L Zhao W. Loss of AIM2 expression promotes hepatocarcinoma progression through activation of mTOR-S6K1 pathway. Oncotarget (2016) 7(24):36185–97. doi: 10.18632/oncotarget.9154 PMC5094992 27167192

[33] Yan Y Zheng L Du Q Yazdani H Dong K Guo Y. Interferon regulatory factor 1(IRF-1) activates anti-tumor immunity *via* CXCL10/CXCR3 axis in hepatocellular carcinoma (HCC). Cancer Lett (2021) 506:95–106. doi: 10.1016/j.canlet.2021.03.002 33689775 PMC8009854

[34] Zhang X Zhang P An L Sun N Peng L Tang W. Miltirone induces cell death in hepatocellular carcinoma cell through GSDME-dependent pyroptosis. Acta Pharm Sin B (2020) 10(8):1397–413. doi: 10.1016/j.apsb.2020.06.015 PMC7488361 32963939

[35] Ding G-Y Zhu X-D Ji Y Shi G-M Shen Y-H Zhou J. Serum PON1 as a biomarker for the estimation of microvascular invasion in hepatocellular carcinoma. Ann Trans Med (2020) 8(5):204. doi: 10.21037/atm.2020.01.44 PMC7154400 32309351

[36] Zhang F Cui JY Gao HF Yu H Gao FF Chen JL. Cancer-associated fibroblasts induce epithelial-mesenchymal transition and cisplatin resistance in ovarian cancer *via* CXCL12/CXCR4 axis. Future Oncol (2020) 16(32):2619–33. doi: 10.2217/fon-2020-0095 32804554

[37] Huang Y Li L-Z Zhang CZ-Y Yi C Liu L-L Zhou X. Decreased expression of zinc-alpha2-glycoprotein in hepatocellular carcinoma associates with poor prognosis. J Trans Med (2012) 10(1):1–10. doi: 10.1186/1479-5876-10-106 PMC3476987 22625427

[38] Ma H Liang X Chen Y Pan K Sun J Wang H. Decreased expression of BATF2 is associated with a poor prognosis in hepatocellular carcinoma. Int J cancer (2011) 128(4):771–7. doi: 10.1002/ijc.25407 20473897

[39] Wu X Zhang L Zhou J Liu L Fu Q Fu A. Clinicopathologic significance of LAIR-1 expression in hepatocellular carcinoma. Curr problems cancer (2019) 43(1):18–26. doi: 10.1016/j.currproblcancer.2018.04.005 29776595

[40] Wang L Wei Q Zhang M Chen L Li Z Zhou C. Identification of the prognostic value of immune gene signature and infiltrating immune cells for esophageal cancer patients. Int Immunopharmacol (2020) 87:106795. doi: 10.1016/j.intimp.2020.106795 32707495

[41] Ren X Ji Y Jiang X Qi X. Downregulation of CYP2A6 and CYP2C8 in tumor tissues is linked to worse overall survival and recurrence-free survival from hepatocellular carcinoma. BioMed Res Int (2018) 2018:5859415. doi: 10.1155/2018/5859415 30148168 PMC6083600

[42] Ouyang G Yi B Pan G Chen X. A robust twelve-gene signature for prognosis prediction of hepatocellular carcinoma. Cancer Cell Int (2020) 20(1):1–18. doi: 10.1186/s12935-020-01294-9 32514252 PMC7268417

[43] Hu DG Marri S McKinnon RA Mackenzie PI Meech R. Deregulation of the genes that are involved in drug absorption, distribution, metabolism, and excretion in hepatocellular carcinoma. J Pharmacol Exp Ther (2019) 368(3):363–81. doi: 10.1124/jpet.118.255018 30578287

[44] Cao F Luo A Yang C. G6PD inhibits ferroptosis in hepatocellular carcinoma by targeting cytochrome P450 oxidoreductase. Cell Signalling (2021) 87:110098. doi: 10.1016/j.cellsig.2021.110098 34325001

[45] Ku C-Y Liu Y-H Lin H-Y Lu S-C Lin J-Y. Liver fatty acid-binding protein (L-FABP) promotes cellular angiogenesis and migration in hepatocellular carcinoma. Oncotarget (2016) 7(14):18229. doi: 10.18632/oncotarget.7571 26919097 PMC4951284

[46] Bouattour M Mehta N He AR Cohen EI Nault J-C. Systemic treatment for advanced hepatocellular carcinoma. Liver cancer (2019) 8(5):341–58. doi: 10.1159/000496439 PMC6873089 31768344

[47] Hage C Hoves S Strauss L Bissinger S Prinz Y Pöschinger T. Sorafenib induces pyroptosis in macrophages and triggers natural killer cell–mediated cytotoxicity against hepatocellular carcinoma. Hepatology (2019) 70(4):1280–97. doi: 10.1002/hep.30666 31002440

