# Supplementary material for: A pyroptosis-related gene signature for prognosis prediction in hepatocellular carcinoma
Source: Front Oncol. 2023 Mar 27;13:1085188. doi: 10.3389/fonc.2023.1085188 (PMC10084936; doi:10.3389/fonc.2023.1085188)

Supplementary Materials

1. **Supplementary Table 1**
2. **Supplementary Table 2**
3. **Supplementary Table 3**
4. **Supplementary Table 4**
5. **Supplementary Table 5**
6. **Supplementary Table 6**
7. **Supplementary Figure 1**

Supplementary Table S1. Detailed List of Pyroptosis Genes

Gene List
AIM2 APIP BAK1 BAX CASP1 CASP3 CASP4 CASP5 CASP8 CHMP2A CHMP2B CHMP3 CHMP4A CHMP4B CHMP4C CHMP6 CHMP7 CYCS DHX9 ELANE GSDMA GSDMB GSDMC GSDMD GSDME GZMA GZMB HMGB1 IL18 IL1A IL1B IRF1 IRF2 NAIP NLRC4 NLRP1 NLRP9 TP53 TP63 ZBP1

Supplementary Table S2. Clinical cohorts analyzed in this study

**Charcteristics**	**TCGA**	**ICGA**	**GSE14520**	**GSE10140**	**CPTAC**
total number	363	232	242	80	159
**Age**					
⩽65	216	90	216	-	19
>65	147	142	26	-	140
**Sex**					
Female	118	61	31	-	31
Male	245	171	211	-	128
**TNM stage**					
I	170	36	96	-	91
II	84	106	78	-	14
III	81	71	51	-	52
IV	4	19	-	-	2
**BCLC stage**					
0	-	-	20	-	-
A	-	-	152	-	68
B	-	-	24	-	52
C	-	-	29	-	39
**AFP**					
⩾ 400 ng/ml	63	-	110 (⩾300)	-	101
< 400 ng/ml	213	-	128 (<300)	-	58
**HBV/HCV infection**					
Yes	162	-	-	-	**159**

No	201	-	-	-	**0**
**Cirrhosis**					
Yes	-	-	223	-	112
No	-	-	19	-	47

Supplementary Table S3. Univariable and multivariable analysis of OS in TCGA cohorts

**TCGA cohort**	Overall survival			
	Univariable analysis		Multivariable analysis	
Parameter	HR (95%CI)	P value	HR (95%CI)	P value
TNM stage: III-IV vs I-II	2.49 (1.82-3.40)	<0.001	2.10 (1.44-3.08)	<0.001
Risk score: high vs low	2.57 (1.90-3.47)	<0.001	2.82 (1.89-4.20)	<0.001
HBV/HCV infection: Yes vs No	1.82 (1.36-2.44)	0.001	1.96 (1.33-2.88)	0.001
TMB-High vs TMB-Low	1.91 (1.39-2.62)	0.001	1.80 (1.20-2.71)	0.005
TP53 Mut vs WT	1.40 (1.02-1.92)	0.080		
Asian race: No vs Yes	1.31 (0.96-1.79)	0.155		
Child Pugh: B/C vs A	1.62 (0.89-2.92)	0.183		
Age: ≤65 vs >65	1.23 (0.93-1.67)	0.225		
Sex: male vs female	0.83 (0.61-1.11)	0.292		
Fibrosis score: 5-6 vs 0-4	0.83 (0.52-1.30)	0.489		
Grade 3-4 vs 1-2	1.12 (0.83-1.52)	0.539		
AFP>=400 vs <400	1.06 (0.70-1.60)	0.827		

Supplementary Table S4. Univariable and multivariable analysis of OS in ICGC (LIRI-JP) cohorts

ICGC cohort (LIRI-JP)	Overall survival			
	Univariable analysis		Multivariable analysis	
Parameter	HR (95%CI)	P value	HR (95%CI)	P value
Risk score: high vs low	1.18 (1.12-1.25)	<0.001	1.19 (1.12-1.26)	<0.001
TNM stage: III-IV vs I-II	2.38 (1.30-4.36)	0.005	2.14 (1.12-4.09)	0.211
Sex: male vs female	0.52 (0.28-0.97)	0.039	0.30 (0.15-0.59)	0.001
Age: ≤65 vs >65	1.23 (0.66-2.30)	0.509		

Supplementary Table S5. Univariable and multivariable analysis of OS in GSE14520 cohorts

**GSE14520 cohort**	Overall survival			
	Univariable analysis		Multivariable analysis	
Parameter	HR (95%CI)	P value	HR (95%CI)	P value
TNM stage: III-IV vs I-II	3.64 (2.51-5.27)	<0.001	3.20 (1.69-6.07)	<0.001
Risk score: high vs low	2.11 (2.98-1.49)	<0.001	1.70 (1.08-2.69)	0.023
Tumor size: ≥5cm vs <5cm	1.92 (1.37-2.69)	0.002	1.08 (0.53-1.63)	0.794
AFP: ≥300ng/ml vs <300ng/ml	1.69 (1.20-2.37)	0.011	1.21 (0.77-1.90)	0.399
Cirrhosis: yes vs no	5.09 (1.57-16.51)	0.023	3.02 (0.72-12.52)	0.128
Multinodular : yes vs no	1.65 (1.14-2.39)	0.025	1.24 (0.71-2.15)	0.445
Sex: male vs female	1.86 (1.01-3.41)	0.093		
Age: ≤65 vs >65	1.38 (0.75-2.53)	0.386		
ALT: ≥50UL vs <50UL	1.06 (0.70-1.60)	0.483		

Supplementary Table S6. Univariable and multivariable analysis of OS in CPTAC cohorts

**CPTAC cohort (transcriptomes)**	Overall survival			
	Univariable analysis		Multivariable analysis	
Parameter	HR (95%CI)	P value	HR (95%CI)	P value
TNM stage: III-IV vs I-II	2.69 (1.86-3.91)	<0.001	2.48 (1.54-3.98)	<0.001
Risk score: high vs low	2.99 (1.67-5.35)	<0.001	1.59 (1.02-2.49)	0.042
ALT: >40 vs ≤40	1.66 (1.15-2.40)	0.024	1.36 (0.82-2.23)	0.227
gmma-GT: >40 vs <=40	1.72 (1.12-2.66)	0.039	1.12 (0.61-2.06)	0.705
TB: >17.1 vs <=17.1	2.27 (1.12-2.72)	0.054		
Age: ≤65 vs >65	1.61 (0.84-3.13)	0.229		
AFP: ≥400ng/ml vs <400ng/ml	1.31 (0.89-1.92)	0.253		
Sex: male vs female	1.86 (1.01-3.41)	0.093		

Supplementary Figure 1. Before and after batch-effect removal of the training set (A) and the validation set (B).

A

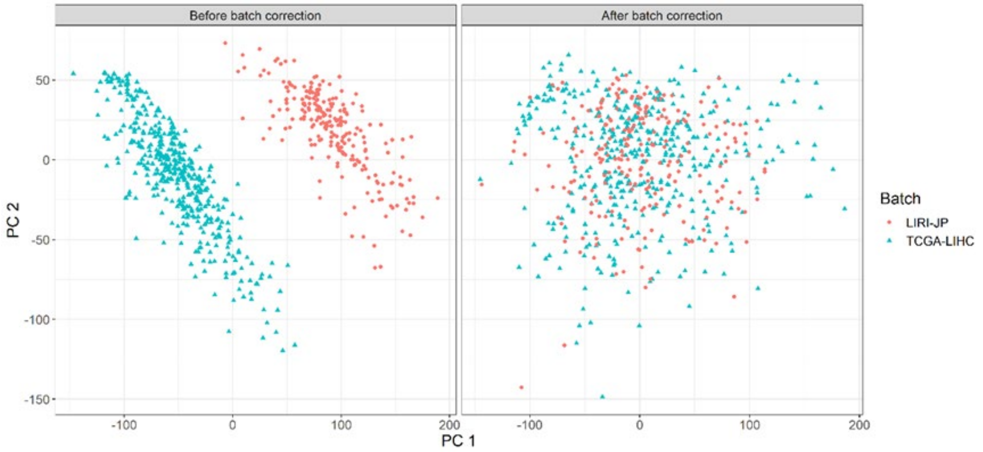

B

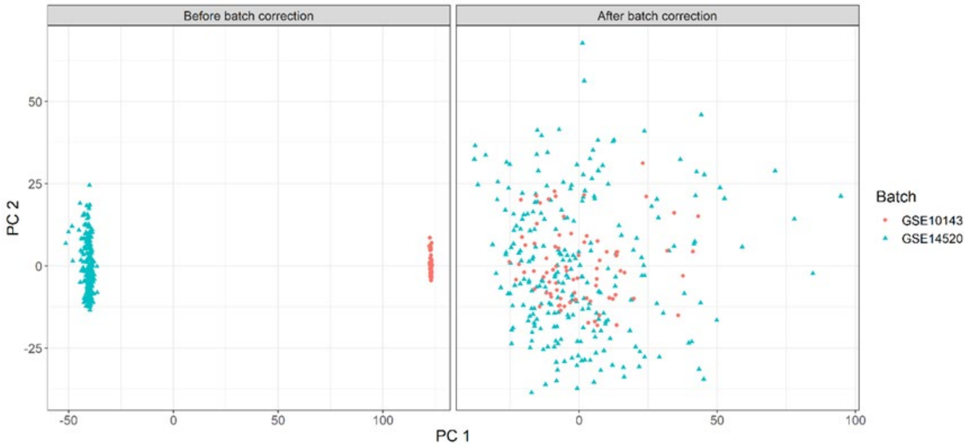

Supplement: Supplementary file 1 [file DataSheet_1.pdf]
